# Elementary School Students’ Subjective Well-Being Before and During the COVID-19 Pandemic: A Longitudinal Study

**DOI:** 10.1007/s10902-022-00537-y

**Published:** 2022-05-11

**Authors:** Ricarda Steinmayr, Patrick Paschke, Linda Wirthwein

**Affiliations:** TU Dortmund, Emil-Figge-Str. 50, Dortmund, 44227 Germany

**Keywords:** COVID-19 pandemic, Subjective well-being, Life satisfaction, Affect, Children

## Abstract

First empirical results indicate that the COVID-19 pandemic has a negative impact on adolescents’ and adults’ subjective well-being. In the present study we focus on the subjective well-being of elementary school children before and after the first pandemic-related school lockdown and examine if possible declines in subjective well-being are especially pronounced for some groups, considering socio-economic status, migration background, and gender as moderators. We tested *N* = 425 elementary school students (mean age: *M* = 8.19; *SD* = 1.04) longitudinally with four measurement points (three before the school lockdown and one after) regarding their general life satisfaction, mood, and domain satisfaction regarding peers, family, and school. Piecewise growth curve models revealed a significant decline in positive mood and in satisfaction with the family. Decline in life satisfaction and satisfaction with peers nearly missed significance. The investigated moderators had no impact on the changes in subjective well-being. We conclude that the pandemic had detrimental effects on young children’s subjective well-being.

**Supplementary Information:**

The online version contains supplementary material available at 10.1007/s10902-022-00537-y.

## Introduction

First studies show that the COVID-19 pandemic as well as the associated infection control interventions implemented by the governments significantly reduced mental health and psychological well-being in the population (e.g., Pappa et al., 2020; Schwinger et al., 2020; Wang et al., 2020). Especially parents with school-aged children and children in care facilities reported a significantly reduced mental health and well-being after the beginning of the pandemic (Patrick et al., 2020). In this context, especially families with hardships related to the pandemic (such as job loss) showed an increase in negative mood (Gassman-Pines et al., 2020). However, less is known about the subjective well-being (SWB) of school students during the pandemic. Especially children and adolescents might suffer from the contact restrictions during the infection control interventions (e.g., school closures). As in particular social relationships are important determinants of SWB in childhood and adolescence (Konu et al., 2002) and school students had hardly any opportunity for social exchange during the pandemic and the school lockdowns, thus mental health and SWB should have decreased significantly. First longitudinal studies indeed demonstrate a decline in life satisfaction and related variables in adolescents (e.g., Magson et al., 2021). However, to our best knowledge, we are not aware of any longitudinal study that tests SWB in elementary school children before and after the onset of the pandemic. The aim of the present study was to close that research gap. The present study examined self-report data from elementary school children by using a longitudinal design comparing SWB data three times before and once after the school lockdown.

### Subjective Well-Being

The terms “mental health” or “subjective well-being” are not used homogeneously throughout the existing studies addressing the effects of the pandemic. Whereas mental health indicators primarily focus on different mental illnesses such as depression or anxiety, other studies rather refer to “stress” (Jones et al., 2021). Hence, these heterogeneous conceptualizations make it difficult to compare the results of different studies. Moreover, the term “subjective well-being” is also operationalized quite heterogeneously. Basically, two theoretical approaches are mentioned in the literature regarding the construct of SWB. The hedonistic perspective comprises individuals’ cognitive evaluations of their lives as a whole (i.e., global life satisfaction) and reports on affective well-being such as positive and negative emotions (Diener et al., 1999). Moreover, different domain satisfactions are distinguished such as satisfaction with one’s family or peers (Haranin et al., 2007; Long & Huebner, 2014). According to the eudaimonic perspective, well-being occurs when an individual lives in congruence with his or her subjective beliefs. In this context, SWB is measured multidimensionally (e.g., Ryff & Keyes, 1995). In the current manuscript, we refer to the hedonistic perspective of SWB analyzing global and domain specific satisfaction as well as an affective component (positive mood). Besides the theoretical distinction, both cognitive and affective components are empirically distinguishable even though they are substantially related (Diener et al., 2018). This structure of hedonic SWB seems to be comparable for adults and children (Long, & Huebner, 2014).

### Determinants of Subjective Well-Being

If one is interested in examining the effects of circumstances such as a global pandemic on SWB, it is important to review the determinants and correlates of children’s or adolescents’ SWB (e.g., Huebner, 2004). Whereas demographic variables such as gender or objectively achieved success criteria are not or only slightly related to SWB, other variables, such as personality, quality of social relationships, and the subjective perception of one’s life circumstances, display medium to high relationships with SWB (Diener et al., 2018; Meulemann, 2000; Steel et al., 2008). Furthermore, environmental factors such as the family and school environment seem to have an influence on SWB (Shek & Liang, 2018), which is in line with assumptions made by ecological system theories (Bronfenbrenner, 1986). Not especially focusing on children and adolescents, Lyubomirsky et al. (2005) suppose in their model of sustainable happiness that besides personality/genetic factors circumstances in life (e.g., origin or demographics, but also life events) play an additional role of about 10% contributing to overall happiness.

To further evaluate the effects of circumstances such as a pandemic on SWB, one can refer to the research on the relevance of life events on SWB (Luhmann et al., 2012; Lyubomirsky et al., 2005). Studies on the impact of major negative life events on SWB demonstrate that SWB seems to be relatively stable (Diener et al., 1999). However, some negative life events (i.e., unemployment, divorce) might have a small to medium impact on changes in SWB in adults (Luhmann et al., 2012). However, less is known about the impact of life events on children’s and adolescents’ SWB, although there are hints that some life events such as relocations and school changes might have long-term negative effects (Montserrat et al., 2015). One can assume that the COVID-19 pandemic might serve as a negative life event for children and adolescents, as their environment during the pandemic changed considerably. In this context, the stage-environment fit approach by Eccles et al. (1993) suggests that the environment has to fulfill certain prerequisites so that a child develops in a healthy way. The life of school-aged children in Germany, where the data of the current study was collected, changed substantially after the beginning of the COVID-19 pandemic. Schools closed for at least 2 months in spring 2020 and distant teaching was conducted, which was predominantly realized by sending paper–pencil tasks to the students without personal communication or feedback (cf. Steinmayr et al., 2021). Moreover, cultural and sports facilities were closed. Everybody was advised to reduce social contacts outside family as much as possible, and personal meetings were only allowed for persons of max. two households. Thus, the environmental preconditions for a healthy development fostering SWB, such as becoming autonomous, experiencing competence and social relationships (cf. self-determination theory, Ryan & Deci, 2000) were probably not met due to the school closures and social contact restrictions during the COVID-19 pandemic, resulting in a possible decrease in SWB. Indeed, a cross-sectional study conducted during the first lockdown with a representative sample of children and adolescents in Germany demonstrated that two third of them reported to be highly burdened due to the pandemic (Ravens-Sieberer et al., 2021). Furthermore, more children and adolescents reported psychological and health problems compared to a different representative sample tested before the pandemic. Thus, there are theoretical and empirical aspects indicating an impact of the COVID-19 pandemic on SWB.

### COVID-19 Pandemic and Subjective Well-Being

There is little previous research on the effects of other epidemic outbreaks on SWB, especially regarding children. Studies on the Severe Acute Respiratory Syndrome (SARS) virus outbreak, which predominantly occurred in Asia in the years 2002 and 2003, showed a very large extent of psychological distress for the general public and a decrease in mental health (e.g., Cheng & Tang, 2004). However, mainly patients with the disease or those who had recovered were examined (e.g., Cheng & Wing Wong, 2005).

Up until now, the majority of current studies analyzing the impact of the COVID-19 pandemic conducted with children and adolescents refer to mental health constructs such as depression or anxiety, indicating that mental health has declined since the beginning of the pandemic (Bacikova-Sleskova et al., 2021; Chen et al., 2020; Hawke et al., 2020; Jones et al., 2021; Li et al., 2021a; Ravens-Sieberer et al., 2021; Tang et al., 2020). Furthermore, there are some studies assessing children’s SWB via parent ratings (e.g., Neubauer et al., 2021). Since external assessments are not an optimal indicator of a child's own well-being, it seems particularly relevant to ask children themselves (e.g., Pavot et al., 1991). However, there is a lack of studies that have explicitly addressed cognitive as well as affective measures of SWB examining children during the pandemic. Especially longitudinal studies asking the children about their SWB before and after the pandemic are needed. Two cross-sectional cohort studies from Korea and Shanghai found either no differences in life satisfaction between two different cohorts tested before and after the onset of the pandemic (Choi et al., 2021) or even an increase in life satisfaction for 21% of all students (Tang et al., 2020). However, cross-sectional cohort designs are probably not appropriate for testing the effects of a pandemic. It has to be reassured that both the sample tested after the onset of the pandemic and the comparison group tested before are both representative and do not differ in any aspect related to SWB (e.g., home environment, personality). As it is not possible to control for all hypothetically related variables, it cannot be excluded that other variables than the investigated ones caused differences between the cohorts. This is probably the case in these two studies, as results of longitudinal studies differ from those two studies as described in the following. Unfortunately, longitudinal studies addressing the difference in SWB before and after the beginning of the COVID-19 pandemic are still scarce. Magson et al. (2021) examined a sample of *N* = 248 adolescents longitudinally, 1 year before the onset of the pandemic and 2 months after the government restrictions due to the pandemic. Besides anxiety, distress, and depressive symptoms, the authors also investigated the overall life satisfaction. Magson et al. (2021) found a significant decrease in life satisfaction (*d* = 0.61) from the first to the second measurement point. Among other moderators (e.g., interpersonal conflict or social connectedness), gender significantly moderated the results and girls showed a higher decrease in life satisfaction than boys. Examining *N* = 155 adolescents from the United States of America before and during the beginning of the pandemic at two measurement points, Rogers et al. (2021) found hints for an increase in negative affect and a decrease in positive affect. Furthermore, these changes in affect were associated with an increase in other mental health problems. Romm et al. (2021) used two samples of *n* = 123 American adolescents before the beginning of the pandemic and *n* = 85 after the beginning of the pandemic to analyse risk and protective factors of psychosocial adjustment during the pandemic. The adolescents reported a higher negative affect during the pandemic and a lower negative affect, but no differences regarding the change in life satisfaction.

Even less is known about moderating factors regarding school students’ SWB development. One can assume that the pandemic might not affect all children and adolescents similarily, indicating the need to examine who suffers more than others (Romm et al., 2021). Especially socio-economic inequalities might have a negative impact on a child’s mental health and hence, also on the SWB of children (Li et al., 2021a). In this context, the pandemic might have more detrimental effects on mental health and SWB for children from socio-economically disadvantaged families. Furthermore, a lower parental education seems to be associated with higher mental health problems in their children during the COVID-19 pandemic (Li et al., 2021a). There is some evidence that especially parents with a lower socio-economic status show a higher decrease in SWB during the pandemic (Li et al., 2021b). This loss of parental well-being could also have detrimental effects on their children according to the Spillover-Crossover model (Bolger et al., 1989; Westman, 2002). Furthermore, cross-sectional studies demonstrated that during the first lockdown, parents from low socio-economic/and or migration households as well as boys’ parents reported lower motivation and less learning progress during distant learning (Steinmayr et al., 2021). Besides other intrapersonal factors, gender and socio-economic status were important predictors of individual differences in SWB during the pandemic in large samples of adolescents from different countries (Engel de Abreu et al., 2021). Being female was also a potential risk factor for a decrease in life satisfaction in Australian adolescents during the COVID-19 pandemic (Magson et al., 2021). As girls and female adolescents also show higher expressions regarding anxiety (e.g., Bender et al., 2012), the pandemic might have especially detrimental effects for them. Hence, besides the socio-economic status, it seems to be relevant to additionally include gender as a potential moderator variable regarding the effects of the pandemic on SWB.

Bhogal et al. (2021) also raise concerns that the COVID-19 pandemic might have differential effects on mental health for children from cultural minorities (see also Sneed et al., 2020): Referring on Black American communities in the United States, the authors assumed that the pandemic has specific consequences such as a fear of cultural bias regarding the diagnostic and treatment of COVID-19 or a re-traumatization of already-traumatized humans. Analyzing Austrian adolescents during the COVID-19 pandemic, Pieh et al. (2022) found that the migration background was associated not only with a poorer mental health but also with a lower SWB in children during the pandemic. To our knowledge, there is no study investigating the relevance of a migration background for changes in SWB before and after the beginning of the pandemic longitudinally.

Previous studies regarding mental health and SWB predominantly focus on adolescents because the social distancing interventions and the school closures might be especially problematic for them (e.g., Janssen et al., 2020; Magson et al., 2021). But social isolation and homeschooling could also have detrimental effects for younger children. As younger children need more attention and guidance by their parents regarding their school work (El Nokali et al., 2010) and might have less social contact to other children, they might especially be at risk regarding mental health and SWB. For example, a Chinese study with children younger than six years old found hints that these children were more afraid than older children that family members would be infected by COVID-19 (Jiao et al., 2020). However, no previous study has focused on the general and domain-specific SWB of elementary school children before and during the pandemic so far.

### The Present Research

As social relationships, experiencing autonomy, and being competent are important determinants of SWB, we assumed that due to the contact restrictions during the lockdowns, the lockdown of schools and all sport and cultural facilities where children have the possibility to become more independent from their families and receive feedback strengthening their feelings of competence, and due to fears of the children associated with COVID-19, SWB should also have decreased significantly in children. This has already been demonstrated by first longitudinal studies for adolescents (Magson et al., 2021; Rogers et al., 2021; Romm et al., 2021). In this context, it is especially interesting to see if the SWB has changed more or less with different subgroups. Thus, socio-economic status, migration household, and gender should be considered regarding the aforementioned possible declines (Li et al., 2021a; Magson et al., 2021; Steinmayr et al., 2021). Moreover, especially longitudinal studies are required when examining the change in SWB before and after the onset of the pandemic. Hence, the current longitudinal study aims at analyzing the overall and domain-specific SWB of elementary school children by using self-report data. We examined three measurement points before the beginning of the pandemic and one measurement point after the beginning of the pandemic. We expected a significant decline in life satisfaction, positive mood, and domain satisfactions after the beginning of the COVID-19 pandemic. Furthermore, we exploratively investigated the aforementioned moderators. We assumed a higher decrease in SWB measures for girls than for boys (Engel de Abreu et al., 2021; Magson et al., 2021) and a higher decrease for children from a low socio-economic household (Engel de Abreu et al., 2021; Li et al., 2021a). No assumptions were made for migration background due to missing preliminary longitudinal studies on SWB.

## Method

### Sample and Design

We examined *N* = 425 elementary school students (*n* = 208 girls, *n* = 213 boys, *n* = 4 students did not report their gender) from Grades 2 to 4 from four different schools in Germany. The study is part of a larger project that started before the COVID-19 pandemic (Christiansen et al., 2019) and gave us the unique possibility to longitudinally follow those kids before and during the pandemic. As the study was not planned for the purpose of the present article, we did not conduct a power analysis. Data were assessed across four measurement points (*t*1 through *t*4). *T*1 took place between September and December 2018, *t*2 between May and June 2019, *t*3 between November and December 2019, and *t*4 between May and June 2020. We chose time intervals of roughly 6 months between the measurement points so we could analyze both developmental trajectories and seasonal differences within each school year. However, as noted above, the study was not designed for the purpose of the present article and therefore, the measurement points were not chosen for this particular purpose either.

**Fig. 1:**
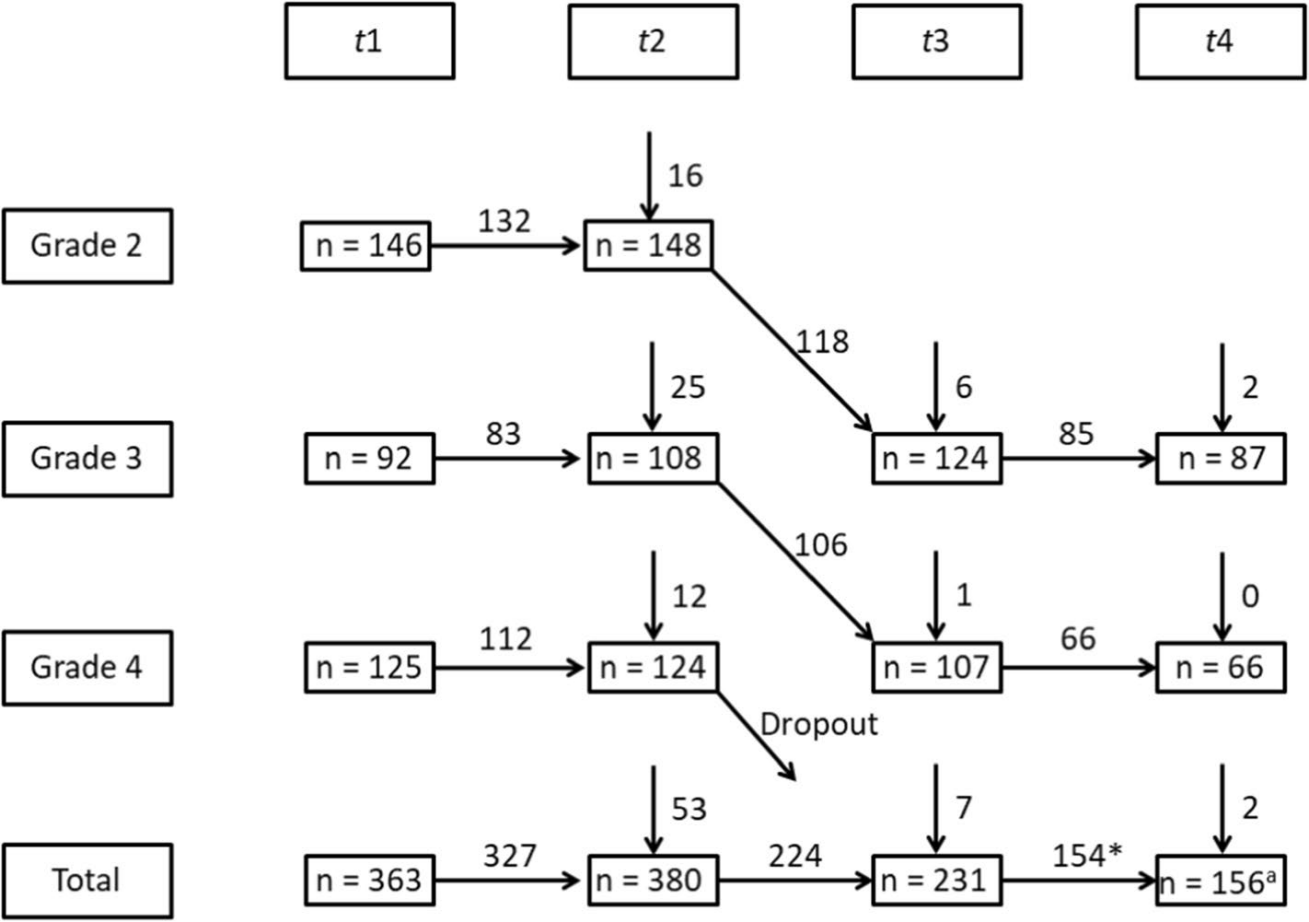
Sample sizes across school grades and measurement time points. *Note.* ^a^The total number of participants at *t*4 is larger than the sum of the number of participants at *t*4 in grades 3 and 4 because not all students reported their grade

Since students in Germany start secondary school after Grade 4, students that were in Grade 4 at *t*1 were only followed until including *t*2, while students that were in Grade 2 or 3 at *t*1 were followed to the end of the study (see Fig. 1 for details on sample sizes). At *t*1, participants were on average 8.19 (*SD* = 1.04) years old. According to official statistics on migration background, we coded migration background in the following way: either the student or one of their parents were not born in Germany or the language spoken most commonly at home was not German (MSW, 2017). Migration background was coded as 1, which applied to 59.3% of all investigated students (38.4% had no migration background and 2.4% gave no information on any variable related to migration background). Thus, the percentage of participants with a migration background was higher than the same percentage for elementary school students in the same federal state in 2016 (41.5%, MSW, 2017) but typical for the region where the study took place (Ruhr-area; cf. Steinmayr et al., 2017).

Across *t*1 through *t*3, measures were administered by trained researchers and research assistants in class settings. At *t*4, due to the COVID-19 pandemic, the measures were administered by the students’ teachers, instead, who received detailed instruction on how to administer the questionnaires. The present study is in accordance with ethical guidelines for psychological research (validated by the ethics committee of the TU Dortmund University). We received informed consent forms from the parents of all participating students as well as the students themselves. At each measurement time point, students were informed that their participation was voluntary, anonymous, and that no one would have access to their personal data.

### Measures

*Subjective Well-Being.* General life satisfaction was assessed with six items from the life satisfaction scale of the Habitual Subjective Well-Being Scale (HSWBS; Dalbert, 1992; e.g., “My life could hardly be happier than it is.”). The internal consistency of this scale across the different measurement time points was high (0.82 < α < 0.85; Table 1). General mood was assessed with two items of the general mood scale of the HSWBS (e.g., “Usually, I feel pretty happy.”). The internal consistency of this scale across the different measurement time points was acceptable to high (0.71 < α < 0.81; Table 1). The items of both scales were rated on a scale from 1 (does not apply at all) to 5 (fully applies).

**Table 1:** Sample sizes (*n*), means (*M*), standard deviations (*SD*), skewness, kurtosis, and Cronbach’s α for all nondichotomous analyses variables across measurement time points (*t*1 through *t*4)

	*n*	*M*	*SD*	Skewness	Kurtosis	α
*T*1						
General life satisfaction	283	4.17	0.81	−1.40	2.21	0.83
General Mood	289	4.14	0.95	−1.26	1.31	0.71
Life satisfaction family	282	4.58	0.62	−2.11	5.12	0.65
Life satisfaction peers	283	4.35	0.91	−1.83	3.01	0.85
Life satisfaction school	279	3.95	1.24	−1.04	0.01	0.87
*T*2						
General life satisfaction	375	4.16	0.86	−1.47	2.08	0.83
General Mood	377	4.15	0.99	−1.29	1.25	0.72
Life satisfaction family	376	4.58	0.61	−2.33	7.12	0.58
Life satisfaction peers	378	4.43	0.81	−2.03	4.61	0.79
Life satisfaction school	373	3.78	1.29	−0.85	−0.38	0.83
*T*3						
General life satisfaction	230	4.16	0.85	−1.37	1.62	0.83
General Mood	230	4.23	0.96	−1.58	2.26	0.81
Life satisfaction family	230	4.64	0.58	−2.98	13.27	0.69
Life satisfaction peers	230	4.45	0.81	−1.89	3.54	0.84
Life satisfaction school	227	3.77	1.31	−0.089	−0.36	0.86
*T*4						
General life satisfaction	157	4.18	0.83	−1.56	2.50	0.85
General Mood	157	4.21	0.96	−1.44	1.77	0.77
Life satisfaction family	157	4.51	0.70	−2.34	6.65	0.74
Life satisfaction peers	157	4.48	0.86	−2.35	5.73	0.89
Life satisfaction school	155	3.79	1.26	−0.92	−0.18	0.94

*Domain-specific life satisfaction.* Student s’ life satisfaction in three domains, namely family (e.g., “My family gets along well together.”), peers (e.g., “My friends are nice to me.”), and school (e.g., “I like being in school.”), was assessed with translated items from the Multidimensional Student’s Life Satisfaction Scale (MSLSS; Huebner et al., 1998). All scales consisted of four items each. However, two of the school specific life satisfaction scales’ items (e.g., “There are many things in school I don’t like.”) were phrased negatively and an inspection of these items revealed that after recoding, they had negative item-scale correlations in Grade 2. Therefore, it seemed that the relatively young students did not understand the negatively phrased items well and consequentially, we removed these items from the scale in all analyses and for all students to ensure comparability. The students rated the items on a scale from 1 (does not apply at all) to 5 (fully applies). Across the four measurement time points, the internal consistencies of the three scales ranged from α = 0.58 to α = 0.74 (family scale), from α = 0.79 to α = 0.89 (peers scale), and from α = 0.83 to α = 0.94 (school scale). Detailed results are reported in Table 1.

*Socio-economic variables.* The number of books present at the students’ home was assessed with the question “How many books are present at your home? Approximately 40 books fit on one bookshelf. Magazines, newspapers, and your schoolbooks are not included.” Students could choose one of six response choices ranging from “fewer than 10” to “more than 500”. We dichotomized the responses into “up to 100” (coded as 0) (55.3%) and “more than 100” (coded as 1) (29.2%) (see Stubbe et al., 2016; Hußmann et al., 2017). We have chosen this indicator of socio-economic status because 1) unlike, for example, parental education, it is easy even for younger students to report and 2) because it is considered a valid indicator of cultural and economic resources at the students’ home (Schwippert, 2019; Schwippert et al., 2020) and is thus commonly used in large scale assessments such as PISA, TIMSS, and IGLU (Hußmann et al., 2017; OECD, 2016; Schwippert et al., 2020; Stubbe et al., 2016).

### Statistical Analysis

*Measurement invariance.* First, we inspected the measurement invariance of the general life satisfaction scale as well as the life satisfaction scales in the family and peer domains across time points to ensure that scales showed comparable psychometric properties across measurement points. Evaluating the measurement invariance of the other two scales was not possible due to the low number of items per scale. We tested four measurement invariance models per scale: configural invariance, metric invariance, scalar invariance, and residual invariance (Putnick & Bornstein, 2016). Non-invariance was indicated by a notable decrease in model fit, that is, by a statistically significant (*p* < 0.05) increase of the Satorra-Bentler corrected χ^2^ in combination with ΔCFI ≥ −0.01, ΔRMSEA ≥ 0.015, or ΔSRMR ≥ 0.030, as recommended by Chen (2007) for testing metric invariance in sample sizes as the one in the present study. As the Chi-Square test is often considered too strict by many authors, a lot only concentrate on the difference in CFI, RMSEA and SRMR, some only with a focus on CFI, RMSEA change (e.g. Siddi et al., 2018). All scales but one reached at least scalar invariance. Satisfaction with family provided somewhat mixed results concerning metric invariance. However, even though constraining factor loadings led to a deterioration of some fit indices, overall model fit was still excellent. An inspection of factor loadings demonstrated that factor loadings slightly differed between measurement points but all items significantly loaded on satisfaction with family at all measurement points (λ ≥ 0.4). For further details on evaluating measurement invariance and the results, see Supplemental Material A.

*Missing data in piecewise growth curve models*. Before computing the models, we inspected the data for missing values and imputed missing values with multiple imputation. Missing values in longitudinal designs can be categorized into resulting from attrition or non-response (Enders, 2010). Across all grades, 36 (9.9%) of participants dropped out between *t*1 and *t*2, 156 (41.1%) between *t*2 and *t*3, and 77 (33.3%) between *t*3 and *t*4 (see Fig. 1). The reasons for the larger number of dropouts at the later measurement points were that (1) between *t*2 and *t*3, the former fourth graders were not followed into secondary school and (2) at *t*4, some classes were split due to the COVID-19 pandemic and not all teachers managed to test twice. In regard to nonresponse, across all measurement time points, 7.6% of the data were missing. We used the mice package in R 4.0.0 to compute multiple imputations of the missing data. Packages such as mice are extensions of the basic program R used to compute certain analyses (e.g., missing data estimation or growth curve models). Supplemental Material B comprises a list of all variables used for missing data estimation and the measurement instruments. We imputed 20 datasets with predictive mean matching, analyzed the models described below for each imputed dataset and pooled the parameter estimates.

*Piecewise growth curve models.* Finally, in order to evaluate whether the COVID-19 pandemic, the outbreak of which happened between *t*3 and *t*4 in Germany, affected students’ SWB, we computed piecewise growth curve models (PGCMs) with the package lme4 in R 4.0.0 (for details, see Kuznetsova et al., 2017). We used the package lmerTest in order to calculate approximate degrees of freedom and *p*-values for the models. PGCMs are an extension to regular growth curve models (GCMs). In GCMs, change over time in a certain variable can be modelled, for example the change in SWB from *t*1 to *t*4. In PGCMs the change across time in more than just one interval can be modelled (Chou et al., 2004), for example the change in SWB from *t*1 to *t*3 and from *t*3 to *t*4. Thus, these models allowed us to compare the linear growth of the dependent variables (e.g., general life satisfaction) before and during the pandemic. Therefore, we specified two linear slopes, one from *t*1 over *t*2 to *t*3 (pre-pandemic) and one from *t*3 to *t*4 (during the pandemic). This was achieved by the computation of PGCMs in which the respective dependent variable was predicted by two time variables (Time1 and Time2) coded as (Time1: *t*1 =  −2; *t*2 =  −1; *t*3 = 0; *t*4 = 0) and (Time2: *t*1 = 0; *t*2 = 0; *t*3 = 0; *t*4 = 1), respectively. Therefore, since both time variables are coded as 0 at *t*3, the intercept represents the dependent variables level at *t*3, while the regression weight of Time1 represents the slope from *t*1 over *t*2 to *t*3 and the regression weight of Time2 represents the slope from *t*3 to *t*4. The effects of the two time variables were estimated as fixed effects and thus, we did not model any variation of the effect of time between subjects. We were not able to also estimate random effects for the two slopes because then the number of parameters to be estimated in the models would have exceeded the number of data points. We did, however, estimate random effects of the intercept dependent on the subject and student’s classes. In other words, while the changes in the dependent variables across time were considered subject-invariant, the basic level of these variables at *t*3 was modelled as variable between subjects and classes. We also added an additional set of predictors to the model: number of books at the students’ home, gender, migration background, and students’ grades. Additionally, we added interaction terms between the two time variables (Time1 and Time2) and number of books, gender, and migration background. This allowed us to test whether changes in the dependent variables over time differ between boys and girls, students with and without migration background, and students with more or less cultural and educational resources at home (number of books).

## Results

### Descriptive Results and Correlations

Sample sizes, means, standard deviations, skewness, kurtosis and Cronbach’s α of all non-dichotomous analysis variables across the measurement time points are reported in Table 1. Since many variables had large absolute values for skewness and/or kurtosis, we computed Kendall’s τ instead of the Pearson correlation coefficient to ensure interpretability of the significance test. We computed Kendall’s τ for the SWB scales within the measurement time points. Table 2 displays the results. At all measurement time points, all SWB scales correlated positively and significantly (0.14 ≤ τ ≤ 0.52; all *p* ≤ 0.01). The found deviation from the normal distribution with a rather positive answer pattern is typical for children of this age as already demonstrated by other studies (cf. Spinath & Spinath, 2005).

**Table 2:** Correlations (Kendall’s τ) between the subjective well-being scales within measurement time points (*t*1 through *t*4)

	*T*1				*T*2			
	2	3	4	5	2	3	4	5
1 General life satisfaction	0.49**	0.34***	0.42***	0.22***	0.46***	0.47***	0.37***	0.25***
2 General mood	−	0.31***	0.41***	0.24***	−	0.35***	0.40***	0.31***
3 Life satisfaction family		−	0.33***	0.14**		−	0.32***	0.28***
4 Life satisfaction peers			−	0.20***			−	0.27***
5 life satisfaction school				−				−

	*T*3				*T*4			
	2	3	4	5	2	3	4	5
1 General life satisfaction	0.50***	0.37***	0.44***	0.21***	0.52***	0.31***	0.39***	0.31***
2 General mood	−	0.37***	0.45***	0.23***	−	0.30***	0.38***	0.39***
3 Life satisfaction family		−	0.39***	0.15**		−	0.36***	0.24***
4 Life satisfaction peers			−	0.22***			−	0.27***
5 life satisfaction school				−				−

*N* = 155–378; **p < 0.01, ***p < 0.001

### Measurement Invariance

Detailed results of the measurement invariance models are reported in Supplemental Material A. For all scales at least configural invariance was demonstrated.

### Piecewise Growth Curve Models

Detailed results for the PGCMs are reported in Table 3. In regard to the main effects of the two time variables, there was no significant slope for Time1 in any model (−0.06 ≤ β ≤ 0.08, all *p* ≥  0.176) and thus, there was no indication that the dependent variables changed significantly between *t*1 and *t*3. On the other hand, the slope for Time2 was significant in the models for general mood (β = −0.20, *p* = 0.032) and life satisfaction in the family domain (β = −0.21, *p* = 0.010). Thus, the value of these variables decreased significantly between *t*3 and *t*4. Additionally, the slope for Time2 narrowly failed to reach significance for general life satisfaction (β = −0.13, *p* = 0.078). The slopes for Time2 for life satisfaction in the peer (β = −0.13, *p* = 0.108) and school (β = −0.05, *p* = 0.576) domains were negative as well, but did not reach statistical significance either. In regard to the main effects of the additional predictors, there was only one significant effect: being male was associated with lower life satisfaction in the school domain (β = −0.28, *p* < 0.001). There were no significant associations of migration background (−0.02 ≤ β ≤ 0.03, all *p* ≥ 0.329), number of books at home (−0.10 ≤ β ≤ 0.03, all *p* ≥ 0.217), or *t*1 grades (−0.10 ≤ β ≤ 0.03, all *p* ≥ 0.129) with the dependent variables. In regard to the interaction effects between the additional predictors and the time variables, two effects narrowly failed to reach significance. First, there was an interaction between Time1 and gender in the model for life satisfaction in the school domain (β = 0.10, *p* = 0.066). Thus, being male was associated with life satisfaction in the school domain declining less between *t*1 and *t*3. Second, there was an interaction between Time2 and migration background in the model for life satisfaction in the family domain (β = −0.05, *p* = 0.079). Thus, having a migration background was associated with declining more in life satisfaction in the family domain between *t*3 and *t*4. However, these two effects were small and did not reach statistical significance. No other interaction reached significance in any model (−0.06 ≤ β ≤ 0.10, all *p* ≥ 0.194).

**Table 3:** Results of the PGCMs predicting change in the subjective well-being scales before SARS-CoV-2 (Time1) and during SARS-CoV-2 (Time2)

	*B*	β	*SE*	*p*
General life satisfaction				
(Intercept)	4.23	0.04	0.063	0.539
Time1	−0.00	0.03	0.062	0.613
Time2	−24	−0.13	0.074	0.078
Books	−0.04	−0.01	0.084	0.948
Time1*Books	−0.01	−0.01	0.062	0.871
Time2*Books	0.09	0.04	0.073	0.553
Sex	−0.15	−0.07	0.074	0.369
Time1*Sex	−0.05	−0.04	0.061	0.469
Time2*Sex	0.20	0.09	0.074	0.207
Migration	0.04	−0.01	0.028	0.744
Time1*Migration	0.06	0.03	0.031	0.413
Time2*Migration	−0.07	−0.02	0.031	0.607
* T*1 Grade	−0.02	−0.02	0.054	0.685
General Mood				
(Intercept)	4.48	0.00	0.054	0.957
Time1	0.07	0.06	0.051	0.261
Time2	−0.49	−0.20	0.088	0.032
Books	−0.11	−0.03	0.078	0.666
Time1*Books	−0.06	−0.05	0.062	0.426
Time2*Books	0.10	0.04	0.079	0.622
Sex	−0.06	0.02	0.067	0.814
Time1*Sex	−0.08	−0.06	0.056	0.296
Time2*Sex	0.10	0.04	0.088	0.652
Migration	0.03	0.01	0.028	0.722
Time1*Migration	0.00	0.00	0.031	0.980
Time2*Migration	−0.01	−0.00	0.034	0.946
* T*1 Grade	−0.10	−0.07	0.049	0.129
Life satisfaction family				
(Intercept)	4.53	0.04	0.054	0.513
Time1	0.04	0.08	0.059	0.176
Time2	−0.24	−0.21	0.078	0.010
Books	0.03	0.03	0.072	0.726
Time1*Books	0.00	0.00	0.070	0.977
Time2*Books	−0.05	−0.03	0.072	0.684
Sex	−0.13	−0.09	0.073	0.235
Time1*Sex	−0.03	−0.04	0.064	0.549
Time2*Sex	0.17	0.10	0.079	0.194
Migration	0.08	−0.00	0.027	0.980
Time1*Migration	0.05	0.03	0.034	0.432
Time2*Migration	−0.18	−0.05	0.031	0.079
*T*1 Grade	0.03	0.04	0.045	0.410
Life satisfaction peers				
(Intercept)	4.52	0.05	0.063	0.386
Time1	0.04	0.05	0.063	0.478
Time2	−0.22	−0.13	0.078	0.108
Books	−0.04	−0.04	0.078	0.611
Time1*Books	0.01	0.01	0.063	0.850
Time2*Books	0.06	0.03	0.070	0.665
Sex	−0.11	−0.08	0.078	0.304
Time1*Sex	−0.02	−0.02	0.065	0.759
Time2*Sex	0.09	0.04	0.081	0.587
Migration	−0.01	−0.02	0.027	0.379
Time1*Migration	0.02	0.01	0.032	0.743
Time2*Migration	−0.08	−0.02	0.031	0.544
*T*1 Grade	−0.02	−0.01	0.051	0.771
Life satisfaction school				
(Intercept)	4.10	0.18	0.069	0.011
Time1	−0.10	−0.06	0.058	0.298
Time2	−0.11	−0.05	0.082	0.576
Books	−0.22	−0.10	0.081	0.217
Time1*Books	−0.08	−0.05	0.069	0.438
Time2*Books	0.10	0.03	0.074	0.664
Sex	−0.24	−0.28	0.075	< .001
Time1*Sex	0.16	0.10	0.056	0.066
Time2*Sex	−0.05	−0.02	0.070	0.799
Migration	0.09	0.03	0.028	0.329
Time1*Migration	0.00	0.00	0.032	0.969
Time2*Migration	−0.06	−0.01	0.035	0.780
*T*1 Grade	−0.05	−0.03	0.058	0.580

*N* = 424. *B* = unstandardized regression weight. β = standardized regression weight. *SE* = standard error; *p* = *p*-value. Time1: *t*1 = −2; *t*2 = −1; *t*3 = *t*4 = 0. Time2: *t*1 = *t*2 = *t*3 = 0; *t*4 = 1. Books: 100 or fewer books hat home = 0; more than 100 books at home = 1. Sex: female = 0; male = 1. Migration: no migration background = 0; migration background = 1

## Discussion

There are no studies dedicated to young children’s self-reports regarding the effects of the COVID-19 pandemic on different components of SWB longitudinally. In the present study, we aimed at filling this gap by using data on the SWB (life satisfaction, positive mood, domain satisfaction) of elementary school children assessed three times before and once during the COVID-19 pandemic. Piecewise growth curve models revealed a significant decrease for general positive mood and life satisfaction in the family domain after the onset of the COVID-19 pandemic. Even though not statistically significant, life satisfaction and satisfaction with peers also declined. We could not confirm neither gender, socio-economic status, nor migration background as a consistent moderator regarding the decrease in SWB.

Several theories posit an impact of life events on SWB. For example, the sustainable happiness model and accompanying studies (e.g. Luhmann et al., 2012; Lyubomirsky et al., 2005) demonstrate that major life events impact on general SWB and domain-specific satisfaction. Furthermore, the self-determination theory claims that the need for autonomy, competence, and relatedness fosters SWB (Deci & Ryan, 2008). In line with these theories, we found that the COVID-19 pandemic had negative effects on positive mood and the satisfaction with the family. Because mood as the affective component of SWB shows high associations with other mental health measures (Headey et al., 1993), our results are consistent with previous studies on adults and adolescents revealing negative effects of the COVID-19 pandemic on depression or anxiety (e.g., Hawke et al., 2020; Jones et al., 2021; Ravens-Sieberer et al., 2021; Schwinger et al., 2020). This decline could be explained by the loss of social relationships during the infection control interventions as close and functioning social relationships are one of the most important determinants of SWB (Deci & Ryan, 2008; Diener et al., 2018; Konu et al., 2002). Furthermore, especially elementary school-parents reported that their children needed quite a lot parental support and indicated that their children learned less during the school lockdown (Steinmayr et al., 2021) which contradicts children’s need for autonomy and competence. According to the self-determination theory, both aspects are important for a person’s SWB (Deci & Ryan, 2008). Indeed, an autonomy-supportive parenting style was associated with their children’s psychological adjustment during the school lockdown (Neubauer et al., 2021). Last but not least, the stage-environment fit approach by Eccles et al. (1993) states that children’s healthy development is only possible if the environment fulfills the prerequisites for a healthy development. During the pandemic environment greatly changed for children with all extra-familiar activities such as school, sports etc. being cancelled. As children need input from different environmental layers for a positive development (Bronfenbrenner, 1979), the prerequisites for positive development were not given for all children during the pandemic (Diener et al., 2018; Konu et al., 2002).

Our study is one of the first studies showing negative effects of the COVID-19 pandemic on the satisfaction with the family. Because of the school lockdown during the pandemic, parents with school-aged children were very burdened and reported a significantly reduced SWB during and after the social distancing interventions implemented by the governments compared to the time before (Patrick et al., 2020). Parents not only had to cope with the contact restrictions and school closures during the infection control interventions but also with the double burden with regard to their work, indicating that parents were exposed to increased stress (Calvano et al., 2021; Li et al., 2021b; Thorell et al., 2021). According to the Spillover-Crossover model (Bolger et al., 1989; Westman, 2002), the parental stress might have influenced their children’s SWB. Furthermore, there are first reports showing that children are more frequently neglected or maltreated during the increased time that children stayed at home during the lockdowns (Griffith, 2020) and hence, this might also have led to a lower level of family satisfaction.

However, the hypotheses regarding the decrease in SWB were just partly confirmed as we found no significant effects for general life satisfaction and the satisfaction with school and with peers did not significantly differ from zero at *p* < 0.05. However, in accordance with our hypotheses, life satisfaction and satisfaction with peers also declined and scarcely missed significance. Several methodological aspects might have led to the fact that life satisfaction slightly missed to become a significant effect in contrast to mood. First, the sample especially at *t4*, thus after the pandemic onset, was rather small. Thus, the effect might have missed statistical significance despite being of large practical relevance. Second, life satisfaction was more stable than mood with less intraindividual change as already demonstrated by other authors (Diener et al., 2018). This causes difficulties in detecting significant interindividual differences in change. A further explanation for the lower change rate in life satisfaction in comparison to mood might be that the mood component comprises emotions that are more prone to change quickly as a reaction to external circumstances than a cognitive construct such as life satisfaction (see Luhmann et al., 2012). Given the fact that we tested children’s life satisfaction about three months after the first lockdown, it might be that the time comprising the pandemic and related changes of our daily life was not long enough to equally impact on mood and life satisfaction. An additional test of the same children after now two years of pandemic and related changes might demonstrate equal impacts on life satisfaction and mood.

As found for life satisfaction, satisfaction with peers also only declined marginally significantly. Again, the small sample size might have prevented the analysis from finding a significant effect. Furthermore, in May 2020, the infection control interventions were slightly reduced by the government in Germany and the children were allowed to attend school again, some in split classes. Thus, the children could meet some of their friends and classmates at school again, and consequently the satisfaction in these domains might have been higher after the school lockdown than during this phase. However, social distancing was still recommended and thus, besides school, children were not able to meet their friends as before the pandemic. This might explain why there was still a marginally significant decrease in satisfaction with peers.

Contrary to the results by Magson et al. (2021) or Li et al. (2021a), we found no hints that socio-economic status or gender were moderators regarding the effects of the pandemic on SWB. Only migration background moderated the decline in students’ satisfaction with one’s family marginally, replicating first findings by Pieh et al. (2022) or Sneed et al. (2020). There are several possible explanations regarding this decline in family satisfaction for children with a migration background. For example, the parents of these children might have experienced more stress during the pandemic than parents from children without a migration background due to feelings of discrimination, belonging to a minority, or social exclusion (Belhadj Kouider et al., 2014; Pieh et al., 2022). This higher parental stress might have had effects on their children’s family satisfaction. Furthermore, acculturation theories refer to life traumatic changes families with an immigrant background already experienced before (e.g., leaving their home country and family) and hence, a pandemic might cause a re-traumatization and a high psychological distress for the families (Shi et al., 2019; Sneed et al., 2020). All other moderations were not close to significance. So, the relatively small sample size cannot explain this finding. Accordingly, our results support the assumption that the pandemic or the associated social contact restrictions had similar effects for all children.

## Limitations

Despite the strengths of our study, there are a few shortcomings to mention. First, the study was not planned to investigate the effects of a pandemic on SWB. As we had already tested the children three times before the pandemic, we provide strong evidence on how the pandemic affected children’s SWB. However, we do not know if the pandemic by itself or the contact restrictions or other pandemic-related changes caused declines in SWB. Second, we just examined children from four German elementary schools via self-reports. As school is important for students’ positive development (Bronfenbrenner, 1979) and differences in school climate are associated with students’ SWB (Steinmayr et al., 2018), it might be that differences between the four schools in school climate or other student-related school activities, such as student–teacher relationship, might have contributed to the negative development in students’ SWB. However, this explanation seems unlikely as schools were closed until shortly before the fourth measurement point, and elementary schools in Germany hardly differed in their distant teaching activities during the first school lockdown (Steinmayr et al., 2021). In accordance with these results, teachers of all participating schools reported that they mostly provided written tasks to their students, randomly gave feedback and communicated with the children. Furthermore, schools did not differ in their pedagogical concepts. However, it cannot be ruled out that school climate fostering variables after the school lockdown differed between the schools. Future studies investigating students’ SWB with a sufficient number of schools could investigate whether school climate variables after school lockdowns also influenced students’ SWB on an individual level.

Third, and despite the teachers’ support, we could not reach all students at *t*4 which resulted in a small sample size at this measurement point. Thus, the investigated moderators might not have been significant as the study lacked the power to detect these effects. Furthermore, it might be relevant for future research to investigate additional moderators and mediators influencing students’ general and domain-specific SWB during the pandemic such as parenting, parent–child discussions, or family conflicts (Magson et al., 2021; Tang et al., 2020). Moreover, as also Romm et al. (2021) concluded, it is crucial to further examine protective factors and factors fostering resilience regarding decreases in SWB such as emotion regulation and coping strategies. Especially certain coping mechanism might help to buffer the negative effects of the pandemic on well-being or quality of life (Shamblaw et al., 2021).

Another limitation refers to the fact that we just examined German children. The respective cultural context might additionally be relevant when examining the effects of the pandemic on SWB (Ruiz et al., 2021), although research especially examining children and adolescents is lacking here. However, it could be that countries with prior experiences of an epidemic such as SARS might cope differently and show a lower change in SWB or mental health.

## Conclusion and Practical Implications

The current study provides valuable insight into the self-reports of young children, indicating that life during the COVID-19 pandemic situation is not only related to a decrease in positive mood but also to a decrease in the satisfaction with the family life and possibly in life satisfaction and satisfaction with peers. Notwithstanding that some groups were especially endangered to experience psychological problem due to the pandemic, such as low SES groups (Ravens-Sieberer et al., 2021), our study gives hints that in general children’s SWB and life satisfaction in some domains declined during the pandemic. These results might help teachers and practitioners such as psychotherapists to engage in activities to help children to overcome the negative impacts of the pandemic. For example, schools might provide trainings fostering SWB such as the one by Suldo and colleagues (2015). Given the importance of SWB for mental health (e.g., Chervonsky & Hunt, 2019) and other important outcomes, it is of societal importance not only to overcome the achievement loss due to the school lockdowns (Engzell et al., 2021) but also to address students’ loss in SWB.

## Supplementary Information

Below is the link to the electronic supplementary material.

Supplementary file1 (DOCX 29 kb)

## Data Availability

The data can be requested from the authors via e-mail.
