# Supplementary material for: Elementary School Students’ Subjective Well-Being Before and During the COVID-19 Pandemic: A Longitudinal Study
Source: J Happiness Stud. 2022 May 11;23(6):2985–3005. doi: 10.1007/s10902-022-00537-y (PMC9091542; doi:10.1007/s10902-022-00537-y)
Supplement: Supplementary file 1 — Supplementary file1 (DOCX 29 kb) [file 10902_2022_537_MOESM1_ESM.docx]

**Supplemental Material A – Method and results of evaluating measurement invariance**

We tested the measurement invariance of the general life satisfaction scale as well as the life satisfaction scales for the family and peer domains. It was not possible to test the measurement invariance of the general mood scale and the life satisfaction scale in the school domain because with only two items, a confirmatory factor analysis (CFA) used to estimate the measurement invariance of these scales would be unidentified. Using the measurementInvariance function of the semTools package in R, we computed multigroup CFAs per scale: 1) a multigroup CFA in which only the factor structure is the same across time points, but all parameters are estimated freely and independently (configural invariance), 2) a multigroup CFA in which additionally, the factor loadings are constrained to be equal across time points (metric invariance), 3) a multigroup CFA in which additionally, the intercepts are also constrained to be equal across time points (scalar invariance), and 4) a multigroup CFA in which additionally, the residual variances are also constrained to be equal across time points (residual invariance). We inspected the differences in the values of the CFA’s fit indices (CFI, RMSEA, and SRMR). Several different cutoff criteria for evaluating measurement invariance have been proposed (Putnick & Bornstein, 2016). We chose a difference in the CFI of .01, a difference in the RMSEA of .015 and a difference in SRMR of .03 for metric invariance and .015 for scalar and residual invariance (Chen, 2007). If one of these cutoff criteria was violated, the assumption of the respective measurement invariance was rejected. We tested the different measurement invariance models in order of increasing parameter restrictions. That is, we first compared configural invariance with metric invariance, then metric invariance with scalar invariance, and finally scalar invariance with residual variance. If a certain invariance model was rejected based on the cutoff criteria described above, all more restricted models were also rejected. For example, if metric invariance was rejected, scalar and residual invariance were rejected, as well. Missing data in the measurement invariance models was handled with full information maximum likelihood estimation (FIML).

Detailed results of the measurement invariance models are reported in Table A1. In summary, for general life satisfaction, residual invariance was accepted (all |ΔCFI| ≤ .008; all |ΔRMSEA| ≤ .13; |ΔSRMR| for metric invariance = .026; all |ΔSRMR| for scalar and residual invariance ≤ .005). On the other and, for life satisfaction in the family and peer domains, all forms of invariance beyond configural invariance were rejected. Additionally, the configural invariance models in both the family and peer domains had acceptable values for the CFI and SRMR but not the RMSEA (family: CFI = .971; SRMR = .024; RMSEA = .095; peers: CFI = .983; SRMR = .022; RMSEA = .120; see West et al., 2012). Because of the high values of the RMSEAs of these models, we computed individual CFAs for these scales as well as the general life satisfaction scale, in order to test the structural validity of the scales. Missing data was estimated with FIML in the CFAs. The results are reported in detail in Table A2 and largely reflected the results for the configural measurement invariance models: the CFIs and SRMRs were generally in an acceptable range, with one exception for the family scale at *t*3 (CFI = .932), while the RMSEAs were not (.932 ≤ CFI ≤ 1.000; .010 ≤ SRMR ≤ .052; .000 ≤ RMSEA ≤ .166). However, an inspection of the factor loadings revealed that almost all of them were in an acceptable range (.338 ≤ λ ≤ .912; see Table A3). Thus, there was no reason to exclude specific items from the scales.

Table A1

*Results of the multigroup CFAs used to inspect the instruments’ measurement invariance*

| Model | χ² (df) | CFI | RMSEA | SRMR | Δχ2 (Δdf) | ΔCFI | ΔRMSEA | ΔSRMR | Decision |
| --- | --- | --- | --- | --- | --- | --- | --- | --- | --- |
| General life satisfaction |  |  |  |  |  |  |  |  |  |
| Configural invariance | 95.99 (36) | .974 | .080 | .032 | - | - | - | - | - |
| Metric invariance | 117.88 (54) | .972 | .067 | .058 | 21.89 (18) | -.002 | -.013 | .026 | Accepted |
| Scalar invariance | 138.01 (69) | .970 | .062 | .061 | 20.13 (15) | -.002 | -.005 | .003 | Accepted |
| Residual Invariance | 174.15 (87) | .962 | .062 | .065 | 36.15** (18) | -.008 | .000 | .005 | Accepted |
| Life satisfaction family |  |  |  |  |  |  |  |  |  |
| Configural invariance | 26.93 (8) | .971 | .095 | .024 | - | - | - | **-** | - |
| Metric invariance | 55.00 (20) | .946 | .082 | .066 | 28.07** (12) | -.025 | -.013 | .042 | Rejected |
| Scalar invariance | 65.30 (29) | .944 | .069 | .071 | 10.29 (9) | -.002 | -.013 | .005 | Rejected |
| Residual Invariance | 102.98 (41) | .904 | .076 | .090 | 37.69*** (12) | -.040 | .007 | .019 | Rejected |
| Life satisfaction peers |  |  |  |  |  |  |  |  |  |
| Configural invariance | 38.21 (8) | .983 | .120 | .022 | - | - | - | **-** | - |
| Metric invariance | 76.22 (20) | .969 | .104 | .082 | 38.01*** (12) | -.014 | -.016 | .060 | Rejected |
| Scalar invariance | 94.98 (29) | .964 | .093 | .085 | 18.76* (9) | -.005 | -.010 | .003 | Rejected |
| Residual Invariance | 143.92 (41) | .944 | .098 | .091 | 48.94*** (12) | -.020 | .005 | .006 | Rejected |

*Note.* General life satisfaction: *N* = 425; *t*1 *n* = 284; *t*2 *n* = 378; *t*3 *n* = 230; *t*4 *n* = 157; Life satisfaction family: *N* = 425; *t*1 *n* = 283; *t*2 *n* = 377; *t*3 *n* = 230; *t*4 *n* = 157; Life satisfaction peers: *N* = 425; *t*1 *n* = 283; *t*2 *n* = 378; *t*3 *n* = 230; *t*4 *n* = 157; **p* < .05; ***p* < .01; ****p* < .001.

Table A2

*Results of the CFAs per scale and measurement time point (t1 through t4)*

|  | *n* | χ² (df) | CFI | *RMSEA [90% CI]* | SRMR |
| --- | --- | --- | --- | --- | --- |
| *t*1 |  |  |  |  |  |
| General life satisfaction | 277 | 25.09 (9)*** | .972 | .080 [.044; .118] | .038 |
| Life satisfaction family | 278 | 1.08 (2) | 1.000 | .000 [.000; .099] | .014 |
| Life satisfaction peers | 277 | 1.79 (2) | 1.000 | .000 [.000; .115] | .010 |
| *t*2 |  |  |  |  |  |
| General life satisfaction | 367 | 43.18 (9)*** | .956 | .102 [.072; .113] | .040 |
| Life satisfaction family | 371 | 2.54 (2) | .996 | .027 [.000; .110] | .019 |
| Life satisfaction peers | 368 | 20.19 (2)*** | .963 | .157 [.100; .223] | .042 |
| *t*3 |  |  |  |  |  |
| General life satisfaction | 225 | 20.45 (9)* | .978 | .075 [.031; .119] | .038 |
| Life satisfaction family | 224 | 14.39 (2)** | .932 | .166 [.093; .252] | .052 |
| Life satisfaction peers | 229 | 11.686 (2)** | .976 | .145 [.073; .231] | .027 |
| *t*4 |  |  |  |  |  |
| General life satisfaction | 153 | 6.11 (9) | 1.000 | .000 [.000; .067] | .023 |
| Life satisfaction family | 156 | 7.28 (2)* | .969 | .130 [.038; .237] | .039 |
| Life satisfaction peers | 154 | 5.70 (2) | .991 | .110 [.000; .220] | .023 |

Note. **p* < .05; ***p* < .01; ****p* < .001.

Table A3

*Factor loadings of the CFAs of subjective well-being scales at different measurement time points (t1 through t4) and standard error in brackets*

|  | *Factor loadings* | | | |
| --- | --- | --- | --- | --- |
|  | *T*1 | *T*2 | *T*3 | *T*4 |
| General life satisfaction item 1 | .830 (.068) | .855 (.059) | .912 (.068) | .860 (.089) |
| General life satisfaction item 2 | .601 (.069) | .723 (.058) | .715 (.073) | .667 (.085) |
| General life satisfaction item 3 | .800 (.049) | .778 (.047) | .900 (.065) | .841 (.066) |
| General life satisfaction item 4 | .675 (.073) | .606 (.062) | .625 (.082) | .625 (.090) |
| General life satisfaction item 5 | .796 (.053) | .824 (0.46) | .950 (.062) | .880 (.074) |
| General life satisfaction item 6 | .703 (.061) | .679 (.057) | .512 (.070) | .689 (.081) |
| Life satisfaction family item 1 | .409 (.069) | .454 (.067) | .338 (.059) | .596 (.085) |
| Life satisfaction family item 2 | .522 (.065) | .447 (.058) | .496 (.066) | .479 (.077) |
| Life satisfaction family item 3 | .498 (.047) | .398 (.043) | .466 (.042) | .684 (.057) |
| Life satisfaction family item 4 | .542 (.058) | .450 (.054) | .580 (.057) | .664 (.076) |
| Life satisfaction peers item 1 | .697 (.051) | .785 (.045) | .758 (.054) | .858 (.059) |
| Life satisfaction peers item 2 | .824 (.049) | .612 (.037) | .675 (.045) | .790 (.057) |
| Life satisfaction peers item 3 | .819 (.064) | .756 (.061) | .750 (.067) | .717 (.072) |
| Life satisfaction peers item 4 | .897 (0.63) | .595 (.051) | .803 (.066) | .876 (.077) |

Note. *N* = 153 – 371. Scale setting was achieved by fixing the variances of the scales to 1.

**Supplemental Material B – List of variables used for the estimation of missing data by multiple imputation in the PGCMs**

Table B

*List of variables used as predictors in the multiple imputation of missing data*

| Construct | Measurement instrument |
| --- | --- |
| *T*1Age | - |
| *T*1 through *t*4 general life satisfaction | Habitual subjective well-being scale (HSWBS; Dalbert, 1992) |
| *T*1 through *t*4 general mood |  |
| *T*1 through *t*4 life satisfaction family | Multidimensional student’s life satisfaction scale (MSLSS; Huebner et al., 1998) |
| *T*1 through *t*4 life satisfaction peers |  |
| *T*1 through *t*4 life satisfaction school |  |
| *T*1 through *t*4 school ability self-concept | Questionnaire for the assessment of social and emotional school experiences (FEES; Rauer & Schuck, 2003) |
| *T*1 through *t*4 social integration in school |  |
| *T*1 through *t*4 class climate |  |
| *T*1 through *t*4 feeling of acceptance from teachers |  |
| *T*1 through *t*4 math intrinsic value | Scale assessing subjective educational task values (SESSW; Steinmayr & Spinath, 2010) |
| *T*1 through *t*4 math utility value |  |
| *T*1 through *t*4 math attainment value |  |
| *T*1 through *t*4 German intrinsic value |  |
| *T*1 through *t*4 German utility value |  |
| *T*1 through *t*4 German attainment value |  |
| *T*1 through *t*4 math ability self-concept | German version of the self-description questionnaire I (SQDI; Arens et al., 2011) |
| *T*1 through *t*4 German ability self-concept |  |
| *T*1 through *t*4 math engagement | School engagement scale (Fredericks et al., 2005) |
| *T*1 through *t*4 German engagement |  |
| *T*1 through *t*4 school test anxiety | German version of the trait anxiety inventory (TAI-G; Hodapp, 1996) |
| *T*1 through *t*4 math test anxiety |  |

Note. The missing values of each dependent variable at each measurement time point were predicted by each variable at each measurement time point reported in this list, except for the same variable across all measurement time points (e.g., missing values of general life satisfaction at *t*1 were predicted by all variables in this list except for general life satisfaction at *t*1 through *t*4).

**References.**

Arens, K. A. , Trautwein, U., & Hasselhorn, M. (2011). Erfassung des Selbstkonzepts im mittleren Kindesalter: Validierung einer deutschen Version des SDQI 1 [Assessing self-concept in middle childhood: Validation of a German version oft he SDQI 1]. *Zeitschrift für Pädagogische Psychologie, 25(2),* 131–144. https://doi.org/10.1024/1010-0652/a000030.

Dalbert, C. (1992). Subjektives Wohlbefinden junger Erwachsener: Theoretische und empirische Analysen der Struktur und Stabilität [Subjective well-being of young adults: Theoretical and empirical analyses of structure and stability]. *Zeitschrift für Differentielle und Diagnostische Psychologie, 13,* 207–220.

Fredericks, J.A., Blumenfeld, P., Friedel, J., & Paris, A. (2005). *"School engagement," What do children need to flourish?: Conceptualizing and measuring indicators of positive development*. New York: Springer Science and Business Media.

Hodapp, V. (1996). The TAI-G: A multidimensional approach to the assessment of test anxiety. In C. Schwarzer, & M. Zeidner (Eds.), *Stress, anxiety, and coping in academic settings* (pp. 95–130). Francke.

Huebner, E.S., Laughlin, J.E., Ash, C. & Gilman, R. (1998). Further validation of the Multidimensional Students` Life Satisfaction Scale. *Journal of Psychoeducational Assessment, 16,* 118–134. https://doi.org/10.1177/073428299801600202.

Putnick, D. L., & Bornstein, M. H. (2016). Measurement invariance conventions and reporting: The state of the art and future directions for psychological research. *Developmental Review, 41*, 71–90. https://doi. org/10.1016/j.dr.2016.06.004

Rauer , W., & Schuck, K.-D. (2003). *Fragebogen zur Erfassung sozialer und emotionaler Schulerfahrungen* (FEESS1-2 / 3-4) [Questionnaire for the assessment of social and emotional school experiences (FEES1-2 / 3-4)]. Göttingen: Hogrefe.

Steinmayr , R., & Spinath, B. (2010). Konstruktion und erste Validierung einer Skala zur Erfassung subjektiver schulischer Werte (SESSW) [Construction and first validation of a scale assessing subjective educational task values (SESSW)]. *Diagnostica, 56(4),* 195-211. https://doi.org./0.1026/0012-1924/a000023.

West, S. G., Taylor, A. B., & Wu, W. (2012). Model fit and model selection in structural equation modeling. In R. H. Hoyle (Ed.), *Handbook of structural equation modeling* (pp. 209–231). Guilford Press.
